# A NIR fluorescent smart probe for imaging tumor hypoxia

**DOI:** 10.1002/cnr2.1384

**Published:** 2021-04-03

**Authors:** Kenneth S. Hettie, Jessica L. Klockow, Eui Jung Moon, Amato J. Giaccia, Frederick T. Chin

**Affiliations:** ^1^ Department of Radiology Stanford University School of Medicine Stanford California USA; ^2^ Department of Otolaryngology ‐ Head & Neck Surgery Stanford University Stanford California USA; ^3^ Department of Radiation Oncology Stanford University School of Medicine Stanford California USA

**Keywords:** bioimaging, glioblastoma, hypoxia, NIR fluorescence, smart probe

## Abstract

**Background:**

Tumor hypoxia is a characteristic of paramount importance due to low oxygenation levels in tissue negatively correlating with resistance to traditional therapies. The ability to noninvasively identify such could provide for personalized treatment(s) and enhance survival rates. Accordingly, we recently developed an NIR fluorescent hypoxia‐sensitive smart probe (**NO**_**2**_ **‐Rosol**) for identifying hypoxia via selectively imaging nitroreductase (NTR) activity, which could correlate to oxygen deprivation levels in cells, thereby serving as a proxy. We demonstrated proof of concept by subjecting a glioblastoma (GBM) cell line to *extreme* stress by evaluating such under *radiobiological* hypoxic (*p*O_2_ ≤ ~0.5%) conditions, which is a far cry from representative levels for hypoxia for brain glioma (*p*O_2_ = ~1.7%) which fluctuate little from *physiological* hypoxic (*p*O_2_ = 1.0‐3.0%) conditions.

**Aim:**

We aimed to evaluate the robustness, suitability, and feasibility of **NO**_**2**_ **‐Rosol** for imaging hypoxia in vitro and in vivo via assessing NTR activity in diverse GBM models under relevant oxygenation levels (*p*O_2_ = 2.0%) within *physiological* hypoxic conditions that mimic oxygenation levels in GBM tumor tissue in the brain.

**Methods:**

We evaluated multiple GBM cell lines to determine their relative sensitivity to oxygenation levels via measuring carbonic anhydrase IX (CAIX) levels, which is a surrogate marker for indirectly identifying hypoxia by reporting on oxygen deprivation levels and upregulated NTR activity. We evaluated for hypoxia via measuring NTR activity when employing **NO**_**2**_ **‐Rosol** in in vitro and tumor hypoxia imaging studies in vivo.

**Results:**

The GBM39 cell line demonstrated the highest CAIX expression under hypoxic conditions representing that of GBM in the brain. **NO**_**2**_ **‐Rosol** displayed an 8‐fold fluorescence enhancement when evaluated in GBM39 cells (*p*O_2_ = 2.0%), thereby establishing its robustness and suitability for imaging hypoxia under relevant *physiological* conditions. We demonstrated the feasibility of **NO**_**2**_ **‐Rosol** to afford tumor hypoxia imaging in vivo via it demonstrating a tumor‐to‐background of 5 upon (i) diffusion throughout, (ii) bioreductive activation by NTR activity in, and (iii) retention within, GBM39 tumor tissue.

**Conclusion:**

We established the robustness, suitability, and feasibility of **NO**_**2**_ **‐Rosol** for imaging hypoxia under relevant oxygenation levels in vitro and in vivo via assessing NTR activity in GBM39 models.

## 1 INTRODUCTION

Oxygen‐deprived tumor tissue can transition toward aggressive phenotypes that promote malignancy and increase resistance to standard treatment regimens such as cytotoxic chemotherapy (CCT) and radiotherapy (RT).^1^, ^2^, ^3^, ^4^, ^5^, ^6^ The ability to noninvasively identify hypoxia in tumor tissue could (i) improve patient prognosis; (ii) afford patient stratification, which would advance efforts toward providing personalized treatment; and (iii) consequently increase the patient survival rates.^7^, ^8^, ^9^ When utilized for the purpose of imaging tumor hypoxia, the non‐optical imaging techniques of magnetic resonance imaging (MRI) and positron emission tomography (PET) suffer from the drawback of reporting on the blood oxygenation levels and providing poor contrast levels (ie, tumor‐to‐background ratios, TBRs, of ≤ ~2.0), respectively, due to, in part, employing non‐smart probes (ie, radiotracers) such as ^18^F‐fluoromisonidazole (^18^F‐FMISO).^10^, ^11^, ^12^, ^13^, ^14^ On the other hand, optical imaging techniques, such as fluorescence imaging, is a noninvasive imaging technique that is well‐suited for imaging tumor hypoxia particularly because such modality affords high sensitivity, high spatiotemporal resolution, and multiplexing capabilities.^15^, ^16^, ^17^, ^18^, ^19^ Other fluorescence optical imaging constructs designed for hypoxia imaging suffer from the drawbacks of photophysical and physicochemical deficiencies that limit their efficacy, such as inherently bearing non‐neutral net charges that could prevent cell membrane translocation, and therefore are ill‐suited for such purpose.^15^, ^20^, ^21^, ^22^, ^23^, ^24^

To overcome such drawbacks, we recently implemented a rational design strategy to develop a hypoxia‐sensitive near‐infrared (NIR) fluorescent smart probe (**NO**_**2**_ **‐Rosol**) that is activatable via bioreductive activation afforded by the nitroreductase (NTR) class of enzymes, whose reductive activity toward the nitro group of nitroaromatic moieties could negatively correlate to oxygenation levels.^25^ **NO**_**2**_ **‐Rosol** is a nitrated congener of THQ‐Rosol, which itself derives from the rosol molecular platform.^26^ As such, **NO**_**2**_ **‐Rosol** contains a xanthene core‐based scaffold that is synthesized from the methyoxybenzene‐based tetrahydroquinoxaline (THQ) moiety beginning with a Friedel‐Crafts acylation using the acyl chloride of 3‐nitrobenzene followed by an intermolecular condensation with resorcinol. **NO**_**2**_ **‐Rosol** demonstrates excellent solubility in water as determined from its (linearly) increasing absorbance value (at 550 nm) and varied increasing concentration between 1 to 30 μM.

**FIGURE 1:**
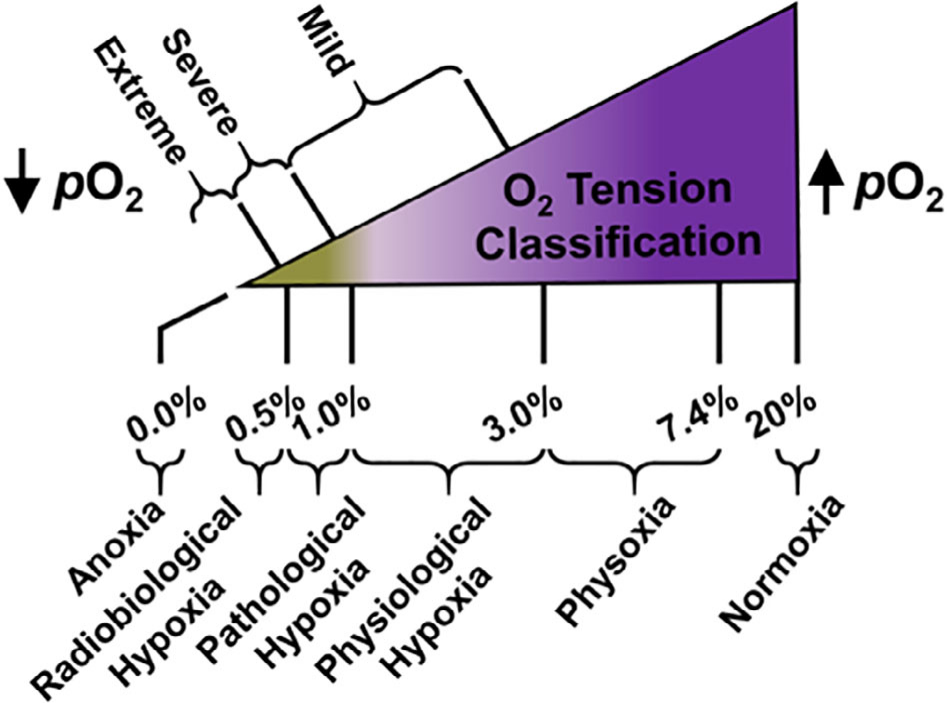
Generalized representation of tumor tissue classification as a function of oxygen tension. Low oxygen tension (hypoxia) criteria are in accordance with measured glioma tumor tissue oxygenation levels (of low‐ and high‐grade including GBM).^27^, ^28^ The unit for a measured oxygenation level is represented by the partial pressure of oxygen (*p*O_2_) expressed as a percentage of total environmental pressure. Figure not drawn to scale

In our previous findings, when compared to a GBM tumor cell line that was propagated under normoxic conditions (*p*O_2_ = 20%) in the same study, our activatable NIR fluorescent smart probe afforded a remarkable 12‐fold OFF‐ON NIR fluorescence enhancement from NTR activity in such cells that experienced *pathological* hypoxic (*p*O_2_ ≤ 1.0%) and *radiobiological* hypoxic conditions (*p*O_2_ ≤ 0.5%) due to such cells being propagated under extreme hypoxic conditions (*p*O_2_ = 0.5%). Pathological hypoxia is defined as the oxygenation level in tumor tissue in which such elicits nonuniform aberrances in its *physiological* (*p*O_2_ = 1.0‐3.0%) hypoxic response that promotes a homeostatic hypoxic environment, whereas radiobiological hypoxia is defined as the oxygenation level in tumor tissue whereby the cytotoxic effect of RT toward such is half maximal (ie, radiation treatment is half as effective due to the hypoxic tumor tissue transitioning to a more radioresistant phenotype) (Figure 1).^4^, ^5^, ^27^, ^28^, ^29^, ^30^, ^31^

As such an extreme state of oxygen deprivation (*p*O_2_ = 0.5%) unrepresentative of typical conditions for hypoxia in glioma tissue in the brain was ideal for efficiently determining the basic functional viability of **NO**_**2**_ **‐Rosol** to selectively undergo bioreductive activation via NTR activity, we found it imperative to extend our earlier work by assessing its ability to afford hypoxia imaging in vitro and in vivo models of patient‐derived GBM tumor cell lines propagated under applicable *physiological* hypoxic (*p*O_2_ = 2.0%) conditions that (i) reflect the typical oxygenation levels in glioma tumor tissue of low‐ and high‐grade in the brain (*p*O_2_ = ~1.7%) and (ii) afford the GBM tumor cell lines to provide homeostatic hypoxic responses. In doing so, we sought to evaluate the robustness and suitability of **NO**_**2**_‐**Rosol** for imaging hypoxia by better mimicking such critical environmental aspects, especially as NTRs can exhibit plasticity in their activity based on oxygenation levels. Importantly, we also sought to establish the feasibility of **NO**_**2**_‐**Rosol** to afford the imaging of tumor hypoxia in vivo via targeting any upregulated NTR activity. In particular, we set out to ascertain if our NIR fluorescent smart probe was capable of promptly diffusing throughout, activating within, becoming sufficiently retained in, and consequently providing effective contrast levels (ie, high TBRs) in *xenograft* murine GBM tumor models post‐administration such that it would successfully afford in vivo imaging of tumor hypoxia via providing effective contrast levels analogous to that which the initial rosol framework also involving a NIR emission‐inducing THQ moiety had allowed for in lymphatic mapping applications (ie, identifying the sentinel lymph node) via NIR fluorescence imaging.^26^, ^32^

Accordingly, herein, we present the evaluation of the robustness, suitability, and feasibility of our NIR fluorescent smart probe for imaging hypoxia in vitro and in vivo utilizing GBM models prepared under relevant oxygenation levels (*p*O_2_ = 2.0%) within *physiological* hypoxic conditions that reflect the typical oxygenation levels of hypoxic glioma tumor tissue found in the brain. Such *physiological* hypoxic conditions afford homeostatic hypoxic responses that if oxygenation levels were more harsh the hypoxic tumor tissue would undergo irreversible dysregulation in its standard processes, and thereby could provide unpredictable non‐homeostatic hypoxic responses with erratic NTR activity that in practice might only rarely occur.^5^, ^33^ Thus, we set forth to initially determine the relative sensitivity of four human‐derived GBM cell lines (GBM U87, GBM U251, GBM2, and GBM39) to oxygenation levels within mild but relevant *physiological* hypoxic conditions (*p*O_2_ = 2.0%) via measuring the total expression levels of the biomarker carbonic anhydrase IX (CAIX) within each cell line and subsequently comparing such to that of the respective GBM cell line propagated under normoxic conditions, wherein CAIX can serve as a surrogate marker for *indirectly* reporting on oxygenation levels and NTR activity by positively correlating to oxygen deprivation levels and positively correlating to NTR activity, respectively.^34^ To *directly* determine whether hypoxia‐induced CAIX total expression levels positively correlated with any upregulated NTR activity when under mild but relevant *physiological* hypoxic conditions (*p*O_2_ = 2.0%), we applied our hypoxia‐sensitive smart probe to the GBM39 cell line for conducting fluorescence microscopy studies due to such GBM cell line having demonstrated relatively the highest hypoxia‐induced upregulated CAIX total expression level of all evaluated GBM cell lines. We next assessed its capability to ultimately afford effective contrast levels (ie, TBRs) upon bioreductive activation by NTR activity post‐administration of **NO**_**2**_ **‐Rosol** to murine GBM39 tumor models by assessing the ability of the NIR fluorescent smart probe to (i) promptly diffuse throughout the tumor tissue upon its administration, (ii) immediately undergo biochemical transformation into its active form, and (iii) maintain a sufficient retention and clearance profile for repeated imaging.

## 2 RESULTS AND DISCUSSION

In our previous work, we evaluated the extent of NTR activity in a GBM cell line (GBM U251) under *extreme* hypoxic (*p*O_2_ = 0.5%) conditions (pathological hypoxic and radiobiological hypoxic conditions), which are grossly atypical hypoxic conditions for glioma tissue in the brain unless under acute additional stress or necrosis.^35^ The environmental stress responses from such cells were elicited by non‐homeostatic hypoxic conditions whose cellular machinery could have been inconsistently operating or simply irretrievably dysregulated, and thus such cells may have not been affording NTR activity that was to be representative of typical hypoxic glioma tumor tissue. As such, any observed NTR activity could have been artificially low or high due to any such dysregulation, and thereby potentially under‐qualifying or over‐qualifying, respectively, the capability for our NIR fluorescent smart probe to render accurate identification (imaging) of hypoxia in glioma tumor tissue (via of detecting NTR activity) that would typically be under mild but relevant *physiological* hypoxic conditions (*p*O_2_ = 2.0%) when hypoxic. Therefore, it was of paramount importance to undertake the studies performed herein and accordingly evaluate such results.

### 2.1 Spectroscopy

**FIGURE 2:**
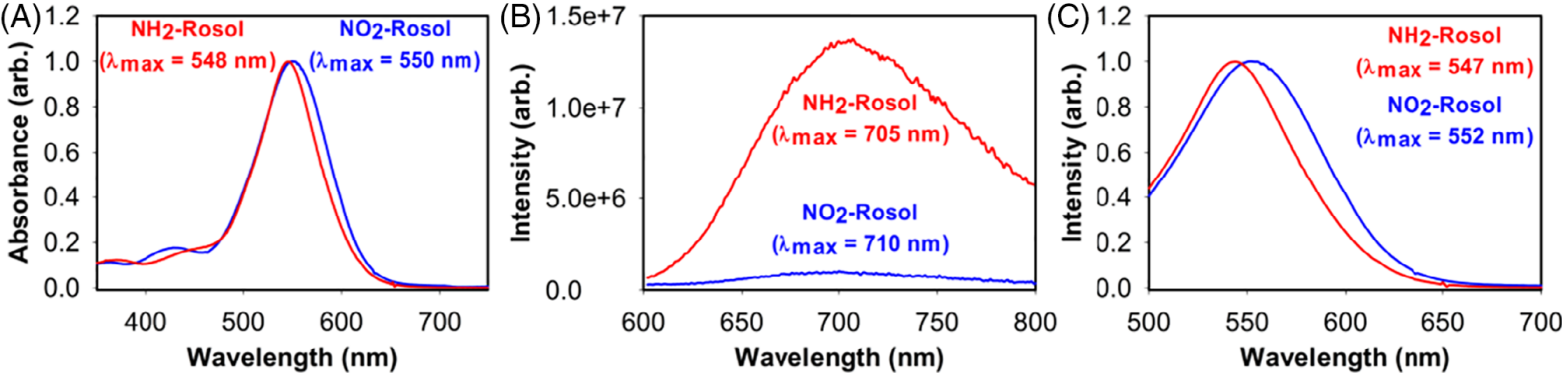
The spectroscopic and photophysical profile of **NO**_**2**_ **‐Rosol** in its inactive and active form (NH_2_‐Rosol). (A) Normalized absorption spectrum. (B) Normalized fluorescence emission spectrum (*λ*_ex_ = 550 nm). (C) Normalized fluorescence excitation spectrum (*λ*_ex_ = 500‐700 nm; *λ*_em_ = 710 nm)

**NO_2_‐Rosol**, in its inactive form, exhibits a measured maximum absorption wavelength and measured maximum fluorescence emission wavelength of 550 and 710 nm, respectively (Figure 2A,B). The active form of **NO**_**2**_ **‐Rosol** (ie, NH_2_‐Rosol) displays a marginal blueshift in its measured maximum absorption wavelength and measured maximum fluorescence emission wavelength with it being at 548 and 705 nm, respectively. The measured maximum wavelength from the excitation spectrum of **NO**_**2**_ **‐Rosol** and NH_2_‐Rosol virtually coincides with that of their absorption profile, which is at 552 and 547 nm, respectively (Figure 2C). Accordingly, the measured extinction coefficient of **NO**_**2**_ **‐Rosol** and NH_2_‐Rosol were determined to be 11 000 and 10 300 M^−1^ cm^−1^, whereby their measured quantum yield was determined to be 0.05% and 2.05%, respectively.

### 2.2 Cellular analyses

**FIGURE 3:**
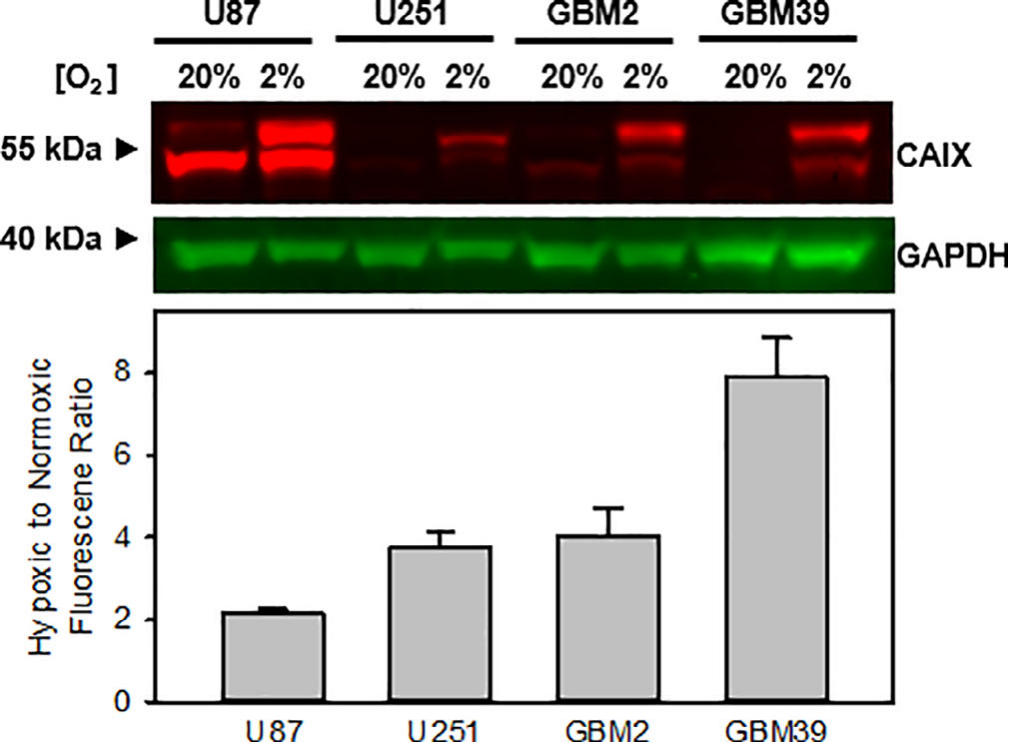
Western blot analysis and semi‐quantification of the hypoxia marker, CAIX, in panel of glioblastoma cell lines. GAPDH is used as a loading control. The GBM cell lines were incubated under normoxic conditions (*p*O_2_ = 20%) or mild but relevant *physiological* hypoxic conditions (*p*O_2_ = 2.0%) for 24 hours prior to cell lysis. CAIX signal was normalized to the loading control and the ratio of hypoxic‐to‐normoxic fluorescence intensity ratio was plotted (*n* = 2, **P < .01 using a one‐way analysis of variance followed by Tukey post hoc tests with the hypoxic‐to‐normoxic intensity of the U87 cells serving as the control)

We initially performed Western blot analyses on whole‐cell lysates from the separate patient‐derived GBM U87, GBM U251, GBM2, and GBM39 cell lines separately incubated under applicable *physiological* hypoxic conditions (*p*O_2_ = 2.0%) and under normoxic conditions (*p*O_2_ = 20%) for 24 hours prior to lysing with RIPA buffer, all in order to determine their relative sensitivity to oxygenation levels via separating, labeling, and semi‐quantifying the total expression level of carbonic anhydrase IX (CAIX). The CAIX biomarker can serve the role of a surrogate marker for *indirectly* assessing NTR activity and both positively correlates to oxygen deprivation levels and can positively correlate to NTR activity.^34^, ^36^, ^37^ CAIX is a zinc metalloenzyme that catalyzes the conversion of carbon dioxide (CO_2_) to bicarbonate (HCO_3_^−^) and H^+^ in the form of the hydronium ion (H_3_O^+^). We stained for CAIX and used glyceraldehyde 3‐phosphate dehydrogenase (GAPDH) as a loading control (Figure 3). CAIX was identified as either one or two bands of approximately 55 kDa in size. Interestingly, we observed one band for the cell lines under normoxic conditions and two bands for the cell lines under *physiological* hypoxic conditions, which has been noted in other literature references in which the higher molecular weight band is considered to be the glycosylated form of expressed CAIX.^38^, ^39^ We observed a relative increase in CAIX total expression level in all cell lines incubated under mild but relevant *physiological* hypoxic conditions (*p*O_2_ = 2.0%) when compared to that of those under normoxic conditions. Interestingly, the CAIX total expression level for the GBM U251 cell line appeared to be in step with that obtained in our previous studies which employed extreme pathological hypoxic and radiobiological hypoxic conditions (*p*O_2_ = 0.5%), whereby a greater extent in oxygen deprivation led to a greater increase in the total expression level of CAIX (~30‐fold when compared to normoxic conditions) in such previous study when compared to that of GBM U251 cells in the current study (~4‐fold when compared to normoxic conditions). Most notable, the GBM39 cell line demonstrated the highest CAIX total expression level under such conditions that represent that of glioma tissue (including GBM) in the brain when compared to that under normoxic conditions. Moreover, the result affords a statistically significant difference, and thus represents reliable data. Given the apparent sensitivity to oxygenation levels, we selected the GBM39 cell line for *directly* evaluating for any correlative NTR activity via employing our NIR fluorescent smart probe both for in vitro imaging studies and subsequently in vivo tumor hypoxia imaging studies.

**FIGURE 4:**
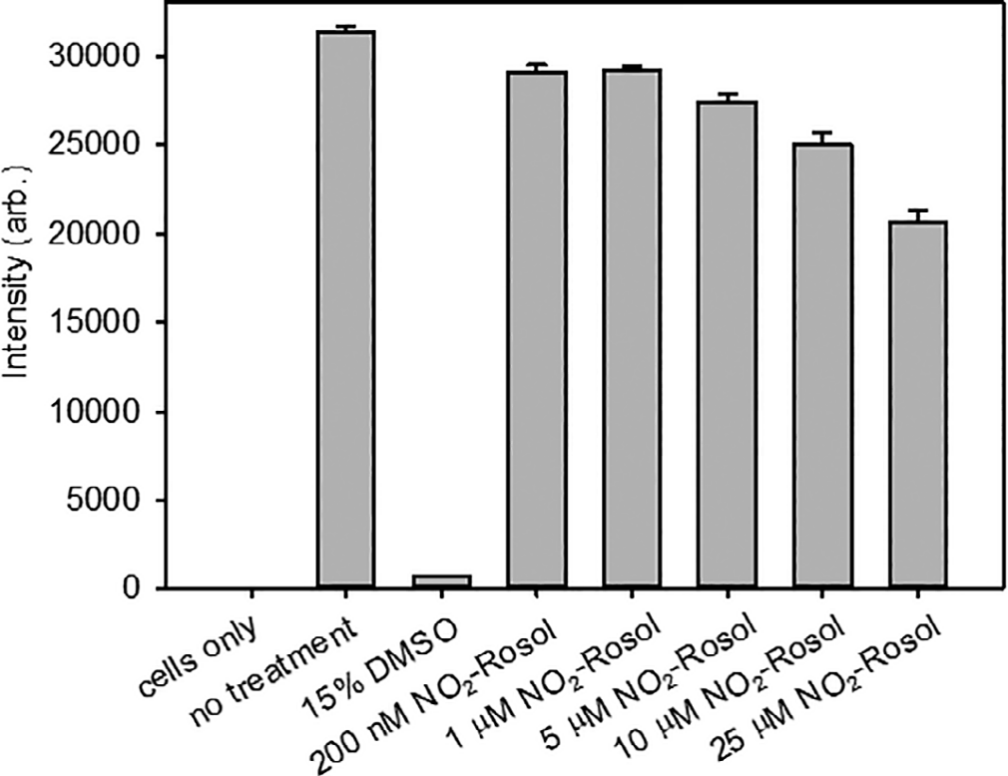
Cell viability assay using varied concentration levels of **NO**_**2**_ **‐Rosol** with GBM39 cells. Cells receiving no treatment were used as a negative control and cells treated with 15% DMSO as a positive control. Cells were stained with Calcein‐AM, a live cell stain, and subsequently the fluorescence intensity was measured at 516 nm (*λ*_ex_ = 494 nm, *n* = 5, *P < .05, ***P < .001, using a one‐way analysis of variance followed by Tukey post hoc tests with the intensity from the cells receiving no treatment serving as the control)

Next, we performed a viability assay to examine the toxicity of **NO**_**2**_ **‐Rosol** to GBM39 cells across a range of relevant concentration levels that we believed would be applicable for use in in vitro and in vivo studies (Figure 4). Cells were incubated at varying concentration levels of **NO**_**2**_ **‐Rosol** and their viability was measured using Calcein‐AM, a live cell stain. The fluorescence intensity was measured on a Tecan plate reader, with higher fluorescence intensity correlating to greater cell survival. After 2 hours of incubating **NO**_**2**_ **‐Rosol** with the GBM39 cells, we observed that the NIR fluorescent smart probe did not pose to be toxic to such cell line at such relevant concentration levels. At all conditions under the varied concentrations of **NO**_**2**_ **‐Rosol**, each result provided a statistically significant difference, which indicates that the results are unlikely due to chance.

### 2.3 Fluorescence microscopy

**FIGURE 5:**
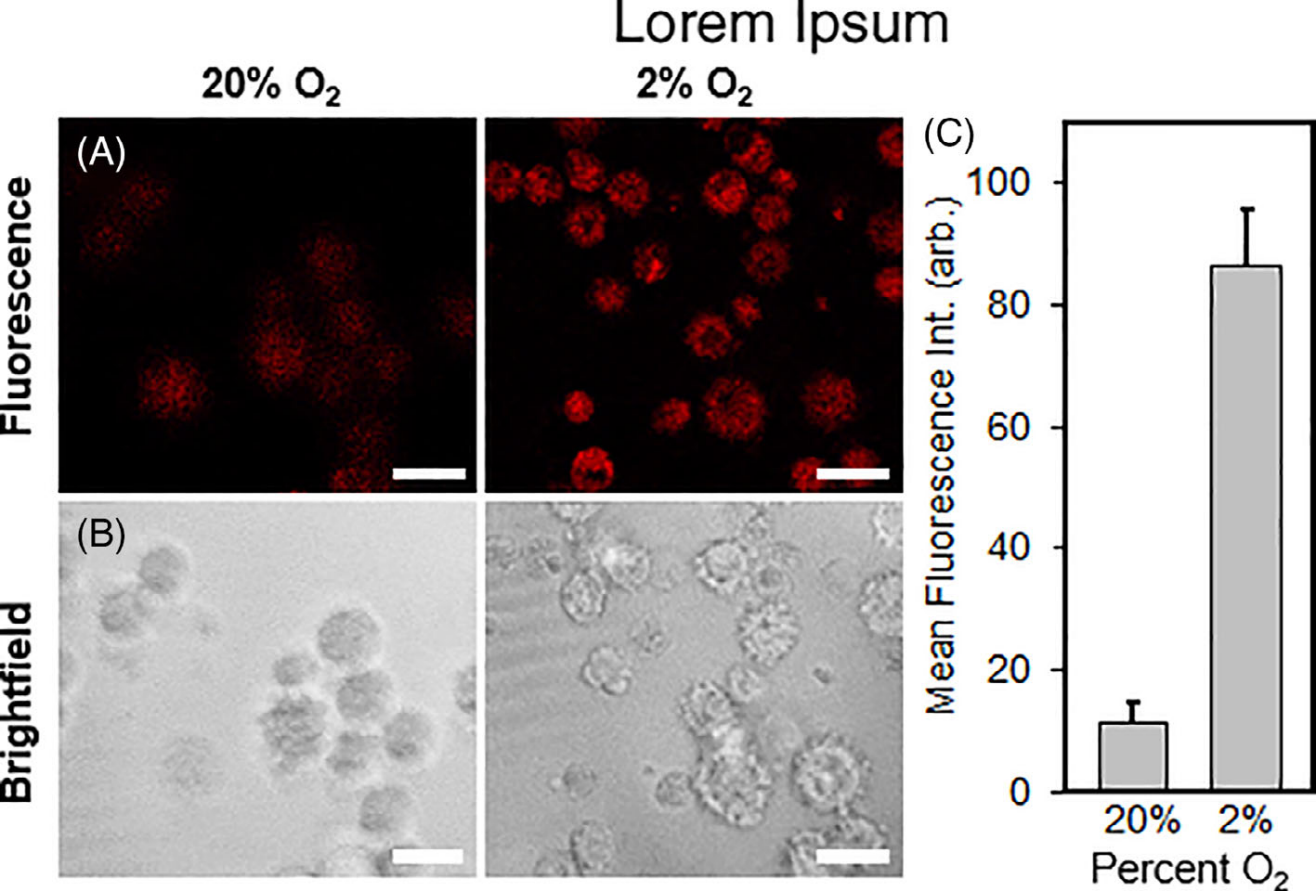
Confocal fluorescence microscopy images of GBM39 cells incubated with **NO**_**2**_ **‐Rosol** (10 μM) under normoxic (20% O_2_) or mild but relevant hypoxic (2% O_2_) conditions. (A) NIR fluorescence imaging. Emission was collected from 650 to 800 nm (*λ*_ex_ = 550 nm). (B) Brightfield imaging. Scale bar = 25 μm. (C) Quantitative analysis of mean fluorescence intensity of the cells (*n* = 50, ***P < .001, by an unpaired *t*‐test with Welch's correction)

We used the GBM39 cell line to *directly* assess for any correlative NTR activity via employing our NIR fluorescent smart probe toward cell imaging studies using confocal fluorescence microscopy (Figure 5). As such, we performed hypoxia imaging experiments in vitro using live GBM39 cells under both mild but relevant *physiological* (*p*O_2_ = 2.0%) hypoxic conditions and under normoxic conditions (*p*O_2_ = 20%). GBM39 cells were seeded onto 35 mm glass‐bottom dishes and subsequently incubated for 24 hours under such oxygenation levels prior to treatment with **NO**_**2**_ **‐Rosol** at 37°C for 20 minutes. Cells were immediately imaged without a wash using a Leica SP8 confocal microscope. We performed confocal microscopy using the 550 nm laser line to excite **NO**_**2**_ **‐Rosol**. As shown in Figure 5, we obtained a significantly higher fluorescence response from **NO**_**2**_ **‐Rosol** when incubated with the GBM39 cells under the mild but relevant *physiological* hypoxic conditions (Figure 5A, upper right panel, *p*O_2_ = 2.0%) compared to that of those under normoxic conditions (Figure 5A, upper left panel, *p*O_2_ = 20%), which indicates the NIR fluorescent smart probe can indirectly report on the oxygenation levels of the cells with the apparent difference in its OFF‐ON NIR fluorescence response (activation) serving as a positive reflection of the upregulated bioreductive activity of NTR. Brightfield images confirmed the presence of healthy cells based on their morphology (Figure 5B, lower panels). Accordingly, **NO**_**2**_ **‐Rosol** displayed a notable 8‐fold fluorescence enhancement when comparing the two different oxygenation level states used in this study, thereby further establishing its robustness and suitability toward imaging *physiological* hypoxia in glioma tumor tissue using distinct cell lines and under various oxygenation levels (Figure 5C). Such results suggested that **NO**_**2**_ **‐Rosol** appeared suitable for potentially affording similarly effective contrast levels upon use in relevant in vivo applications using the GBM39 cell line as well, as the results did afford a statistically significant difference that would lead them to be reliable.

### 2.4 In vivo tumor hypoxia imaging

**FIGURE 6:**
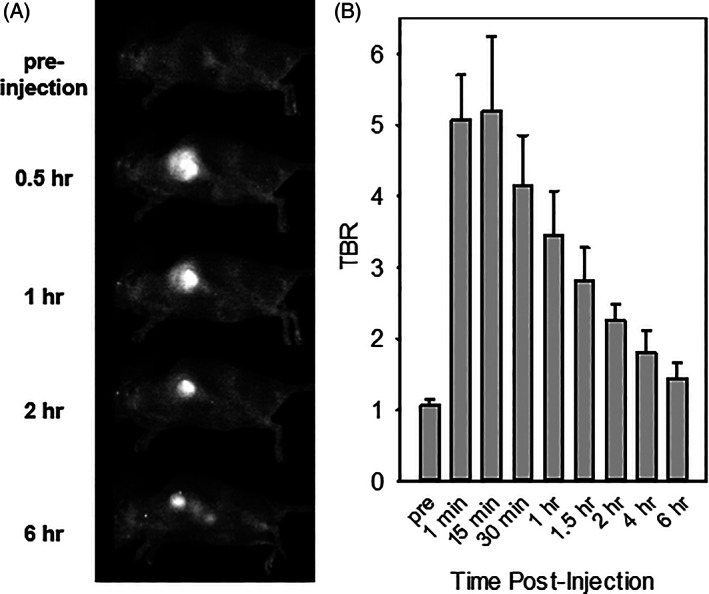
In vivo NIR fluorescence imaging of tumor hypoxia in murine GBM39 tumor model via **NO**_**2**_ **‐Rosol**. (A) NIR fluorescence imaging of hypoxia in murine GBM39 tumor model via the smart probe (25 μM, 50 μL) undergoing bioreductive activation by upregulated NTR activity and subsequently imaged at select time points (*λ*_ex_ = 503‐555 nm). (B) Quantitative analysis from corresponding tumor hypoxia imaging experiment. Bars are expressed as a TBR (*n* = 3). There are no statistically significant differences in the TBR between the time points using a one‐way analysis of variance followed by Tukey post hoc tests with the pre‐injection TBR serving as the control

We assessed the feasibility of **NO**_**2**_ **‐Rosol** to afford in vivo tumor hypoxia imaging via evaluating its NIR fluorescence response elicited by upregulated NTR activity when applied to murine GBM39 tumor models of glioma tumor under presumably such relevant oxygenation levels. More specifically, we evaluated the capability of our NIR fluorescent smart probe to provide effective contrast levels that are superior to those afforded by noninvasive imaging modalities (eg, PET) that employ non‐smart probes (eg, ^18^F‐FMISO) for imaging hypoxic tumor tissue.^13^, ^14^ As the goal of this study was to determine the feasibility of our NIR fluorescent smart probe to afford tumor hypoxia imaging in vivo, we prepared and utilized x*enograft* murine GBM39 tumor models (as opposed to *orthotopic* murine GBM39 tumor models) to limit any potential additional confounding factors, including the blood‐brain barrier (BBB), that might impose toward discerning such at this time especially if we were to not observe a NIR fluorescent response upon initial in vivo evaluation of **NO**_**2**_ **‐Rosol**. The xenograft GBM39 tumor models were prepared by subcutaneously injecting GBM39 cells on the left shoulder of Nu/nu mice, wherein the tumors grew over a period of 6 weeks. The tumors were imaged on a CRi Maestro optical imaging system and the **NO**_**2**_ **‐Rosol** fluorescence response was acquired at select time points (Figure 6). The maximal fluorescence intensity of **NO**_**2**_ **‐Rosol** in its activated form was quickly attained 15 minutes post‐administration due to its successive (i) prompt diffusion throughout, (ii) bioreductive activation elicited by hypoxia‐induced upregulated NTR activity, and (iii) accumulation and retention profile within the hypoxic tumor, all of which afforded a TBR of 5. The efficient combined drainage and hepatic clearance profiles afforded a TBR of 2.5 after 90 minutes as well as nearly restored the initial background fluorescence intensity 6 hours post‐administration, which is a considerably longer retention profile and biological half‐life when compared to other molecular constructs currently used for such purposes. Such longer‐term signal is more ideal for the accurate identification of hypoxia in tumor tissue due to the ability to obtain better image resolution. Importantly, when compared to the TBRs obtained from other noninvasive imaging modalities, such as PET, for the identification of hypoxia in tumor tissue in vivo, the TBRs we obtained from using fluorescence imaging in conjunction with our NIR fluorescent smart probe afforded more than a nearly 3‐fold improvement in contrast levels. Such a higher contrast level affords greater sensitivity in imaging low oxygenation levels.

## 3 MATERIALS AND METHODS

### 3.1 Cell culture

Patient‐derived GBM cell lines (GBM2, GBM39, GBM U87, GBM U251) were used for our studies. GBM2 originated from the Stanford University Hospital & Clinics and was obtained for research purposes after approval from the University's Internal Review Board. We acknowledge GBM39 as a gift from Dr. Sanjiv Sam Gambhir who obtained them from Dr. Paul Mischel (Ludwig Institute for Cancer Research, University of California at San Diego). The GBM39 cells had been transfected with lentiviral vectors that express firefly luciferase to enable bioluminescence imaging. Separately, U87 cells were cultured in DMEM and U251 cells were cultured in RPMI media, each supplemented with 10% fetal bovine serum, 100 U/mL penicillin, and 100 μg/mL streptomycin. GBM2 and GBM39 were grown in a defined, serum‐free media of a 1:1 mixture of Neurobasal‐A Medium (1X) / DMEM/F12 (1X) that also contained HEPES Buffer Solution (10 mM), MEM Sodium Pyruvate Solution (1 mM), MEM Non‐Essential Amino Acids Solution (10 mM, 1X), GlutaMAX‐I Supplement (1X) and Antibiotic‐Antimycotic (1X). All solutions are from Invitrogen/Life Technologies Inc. The full working media also contained h‐EGF (20 ng/mL), h‐FGF‐basic‐154 (20 ng/mL), h‐PDGF‐AA (10 ng/mL), h‐PDGF‐BB (10 ng/mL), and heparin solution, 0.2% (2 μg/mL) as growth factors (all from Shenandoah, Inc.) and B‐27 (Invitrogen/Life Technologies) as supplements. All cells were propagated at 37°C in a humidified atmosphere containing 5% CO_2_ and either 2.0% or 20% O_2_.

### 3.2 Western blot

Four different human‐derived glioblastoma cell lines (U87, U251, GBM2, and GBM39) were each seeded onto a 100 mm culture dish and grown to ~90% confluency. Cells were placed in a hypoxia chamber (Invivo2‐400; Ruskin Technologies, Leeds, United Kingdom), maintained at 2% oxygen for 24 hours, then harvested by trypsinization. Cells were lyzed in ice‐cold Pierce RIPA buffer (Thermo Fisher, USA) containing HALT protease inhibitor cocktail (Thermo Fisher, USA) (~250 μL buffer for 8 million cells). After keeping on ice for 30 minutes and sonicating intermittently, cells were centrifuged at 14000 rpm and 4°C for 15 minutes. The supernatant was saved and the was pellet discarded. Protein concentration was determined by Pierce BCA Protein Assay (Thermo Fisher, USA) as recommended by the manufacturer. Samples containing 60 μg protein, NuPAGE LDS Sample Buffer, and NuPAGE Sample Reducing Agent were heated at 70°C for 10 minutes to reduce the sample and then loaded into each lane of a NuPAGE 4‐12% Bis‐Tris protein polyacrylamide gel, which was electrophoresed at 50 V for 15 minutes then 150 V for 45 minutes under constant voltage. Proteins were transferred to a PVDF membrane by wet blotting methods. Membranes were blocked with 5% BSA/TBST for 1 hour at room temperature and subsequently incubated with antibodies against GAPDH (1:1000, ABClonal) and CAIX (1:200, Santa Cruz Biotechnology) at 4°C overnight. The secondary antibodies were incubated for 2 hours at room temperature and included either a donkey anti‐mouse polyclonal IgG conjugated with Alexa Fluor 680 (1:10000, Abcam) or a mouse anti‐rabbit monoclonal IgG conjugated with CruzFluor 790 (1:10000, Santa Cruz Biotechnology). Blots were visualized using the Odyssey infrared imaging system (LI‐COR; Biosciences, Lincoln, NE). Signal intensities of each blot were quantified using the Odyssey followed by analysis with GraphPad Prism 5 (GraphPad Software, La Jolla, CA).

### 3.3 Synthesis of NO_2_‐Rosol

The methyoxybenzene‐based THQ moiety was prepared according to literature methods.^40^, ^41^ Such moiety (366 mg, 1.13 mmol, 1 eq) was added to a small round bottom flask and dissolved in 20 mL CH_2_Cl_2_, wherein a stir bar was added and the flask was sealed with a rubber septum. The solution was cooled to 0°C and bubbled with N_2_ gas via introducing a continuous gentle stream of N_2_ gas to the solution via a needle alongside a venting needle piercing the septum. The septum was briefly lifted whilst maintaining a strong positive pressure of N_2_ gas and aluminum chloride (904 mg, 6.78 mmol, 6 eq) was added quickly followed by the continuous bubbling of N_2_ gas. Solid 3‐nitrobenzoyl chloride (230 mg, 1.24 mmol, 1.1 eq) was added in a similar fashion followed by the additional bubbling of the mixture with N_2_ gas. Once the solvent volume was reduced by half, the venting needle was removed maintaining a positive N_2_ pressure on the flask. It was sonicated intermittently and allowed to warm to room temperature over 45 minutes. The work‐up involved pouring the reaction mixture over ice and gradually basifying to ~pH 6 with a saturated solution of sodium bicarbonate. The product was extracted in a separatory funnel with CH_2_Cl_2_ (100 mL × 3). The organic layers were combined, dried over magnesium sulfate, and the solvent removed in vacuo. The crude mixture was purified via column chromatography on silica gel. The eluent consisted of 100% methylene chloride which was used to separate out impurities followed by the addition of 1‐5% EtOAc to elute the acylated derivative (80.2 mg, 0.226 mmol, 20%): ^1^H NMR (400 MHz, CDCl_3_) δ 12.73 (s, 1H), 8.52 (s, 1Hz), 8.36 (d, 1H, *J =* 7.6 Hz), 7.99 (d, 1H, *J =* 7.6 Hz), 7.66 (t, 1H, *J =* 8.0 Hz), 6.42 (s, 1H), 6.14 (s, 1H), 3.52 (t, 2H, *J =* 4.8 Hz), 3.43 (q, 2H, *J =* 7.2 Hz), 3.13 (t, 2H, *J =* 4.8 Hz), 3.05 (q, 2H, *J =* 7.2 Hz), 1.24 (t, 3H, *J =* 7.2 Hz), 1.06 (t, 3H, *J =* 7.2 Hz); ^13^C NMR (125 MHz, CDCl_3_) δ 193.4, 161.9, 147.9, 145.4, 141.0, 134.6, 129.5, 127.8, 125.1, 123.8, 112.3, 107.7, 96.6, 48.0, 46.0, 45.5, 45.0, 10.9, 10.1. HRMS calculated for C_19_H_21_N_3_O_4_ (M^+^): 355.1527. Found: 355.1527.

The acylated derivative (147.3 mg, 0.414 mmol, 1 eq), resorcinol (45.6 mg, 0.414 mmol, 1 eq), and methane sulfonic acid (2 mL) were added to a small sealed tube and heated at 95°C overnight. The reaction mixture was poured over ice, basified to ~pH 6 with a saturated solution of sodium bicarbonate and extracted with CH_2_Cl_2_ (100 mL × 3). The organic fractions were combined, dried over magnesium sulfate, and the solvent reduced in vacuo. The crude material was purified via column chromatography on silica gel eluting first with 100% chloroform followed by 85:15 chloroform:MeOH. **NO**_**2**_ **‐Rosol** appeared on the column as a blue band and dried to a blue‐purple solid (40.9 mg, 0.095 mmol, 23%): ^1^H NMR (400 MHz, CDCl_3_) δ 8.43 (d, 1H, J = 8.4 Hz), 8.27 (s, 1H), 7.79 (t, 1H, J = 8.0 Hz), 7.72 (d, 1H, *J =* 7.6 Hz), 6.91 (d, 1H, *J =* 9.6 Hz), 6.64 (dd, 1H, *J =* 1.6, 9.6 Hz), 6.60 (s, 1H), 6.56 (d, 1H, *J =* 1.6 Hz), 5.95 (s, 1H), 3.58 (t, 2H, *J =* 4.8 Hz), 3.52 (q, 2H, *J =* 7.2 Hz), 3.24 (t, 2H, *J =* 4.8 Hz), 3.06 (q, 2H, *J =* 7.2 Hz), 1.30 (t, 3H, *J =* 7.2 Hz), 0.99 Hz (t, 3H, *J =* 7.2 Hz); ^13^C NMR (125 MHz, CDCl_3_) δ 183.4, 158.5, 151.0, 148.3, 146.1, 143.4, 135.9, 135.5, 133.0, 129.8, 128.8, 128.2, 124.4, 124.0, 114.7, 110.9, 104.8, 104.6, 95.6, 47.6, 46.4, 45.3, 44.5, 10.7, 9.5. HRMS calculated for C_25_H_24_N_3_O_4_ (M + H^+^): 430.1761. Found: 430.1759.

### 3.4 Confocal microscopy

GBM39 cells were plated onto 35 mm, 4‐chamber glass‐bottom dishes at a density of 50 000 cells/well and allowed to adhere at 37°C overnight. Cells were incubated in normoxic (20% O_2_) or mild hypoxic (2.0% O_2_) conditions for 24 hours prior to treatment with NIR fluorescent smart probe. **NO**_**2**_ **‐Rosol** (10 μM) was added to the cells and incubated for 20 minutes. Prior to imaging. No washing step was performed. The cells were imaged using a Leica SP8 confocal fluorescence microscope. A 100X (NA = 1.40 OIL) objective lens was used. *λ*_ex_ = 550 nm for **NO**_**2**_ **‐Rosol**. **NO**_**2**_ **‐Rosol** emission was collected between 680 to 900 nm. Pearson's coefficient was calculated using the Fiji (ImageJ) plugin Coloc 2.

### 3.5 Live subject ethical statement

All maintenance, handling, monitoring, and experimental procedures were performed in accordance to a protocol that was approved by The Administrative Panel on Laboratory Animal Care (APLAC) of Stanford University, wherein such approved protocol and committee comply with all federal and state regulations governing the humane care and use of laboratory animals as well as the National Institutes of Health guide for the care and use of laboratory animals (NIH Publications No. 8023, revised 1978).

### 3.6 In vivo tumor models

All animals were anesthetized with inhaled 2‐3% isoflurane for surgical and imaging procedures and recovered with free access to food and water. Eye lubricant and a heating pad were used during anesthetization. For xenograft tumor models, GBM39 tumor cells (0.5 × 10^6^ cells; 150 μL of serum‐free media) were injected subcutaneously into female Nu/nu mice (aged 17 to 18 weeks; Charles River Laboratories) and allowed to grow for 6 weeks. Tumor growth was monitored by calipers and firefly luciferase bioluminescence imaging.

### 3.7 Animal imaging

Bioluminescence imaging was performed by injecting the mice intraperitoneally with firefly D‐luciferin (150 μL, 15 mg/mL in PBS), waiting 10 minutes, and then imaging such using an exposure time of 0.3 second. **NO**_**2**_ **‐Rosol** (25 μM, 50 μL) was injected into the GBM39 tumor models (*n* = 3) and fluorescence imaging was performed on a CRi Maestro spectral fluorescent imager using a 503 to 555 nm excitation filter and 580 nm long‐pass emission filter for spectral cube images or 710 nm for mono‐emission image capture.

### 3.8 Statistical analysis

Unless otherwise noted (and where appropriate), data were expressed as the mean ± SE of the mean (SEM) and analyzed using i) a one‐way analysis of variance, followed by Tukey post hoc tests or ii) an unpaired *t*‐test with Welch's correction, from GraphPad Prism 5 (GraphPad Software, La Jolla, CA).

## 4 CONCLUSION

Here, we evaluated and validated the robustness, suitability, and feasibility of our NIR fluorescent smart probe for imaging hypoxia in vitro and in vivo by assessing the sensitivity of our smart probe to hypoxic conditions and the reflected NTR activity for a panel of GBM models that were under mild but relevant oxygenation levels (*p*O_2_ = 2.0%) within *physiological* hypoxic conditions effectively mimicking the typical oxygenation levels of hypoxic glioma tumor tissue in the brain (*p*O_2_ = ~1.7%), whereby a homeostatic hypoxic environment is particularly crucial for determining the suitability (ie, practical operational range) of **NO**_**2**_ **‐Rosol** due to the plasticity in NTR activity. In measuring the total expression level of CAIX in all four patient‐derived cell lines, we observed the GBM39 cell line to demonstrate the relatively highest CAIX total expression level under mild but relevant *physiological* hypoxic conditions (*p*O_2_ = 2.0%) that represent that of glioma tissue (including GBM) in the brain. As such, any correlative NTR activity was *directly* evaluated in the GBM39 cell line via performing cell imaging studies and subsequently in vivo tumor hypoxia imaging studies in conjunction with employing our recently developed NIR fluorescent smart probe. **NO**_**2**_ **‐Rosol** displayed a marked 8‐fold fluorescence enhancement when such was evaluated in the GBM39 cells (*p*O_2_ = 2.0%) in comparison to that of those under normoxic conditions (*p*O_2_ = 20%), which bolstered its capability for being robust and suitable for imaging *physiological* hypoxia in glioma tumor tissue across different cell lines and at different oxygenation levels. Lastly, we examined the feasibility of **NO**_**2**_ **‐Rosol** to afford effective contrast levels post‐administration to xenograft murine GBM39 tumor models. Indeed, the NIR fluorescent smart probe demonstrated excellent contrast levels via displaying a TBR of 5 upon its activation by NTR in, retention within, and timely combined drainage and clearance from the GBM39 tumor tissue. Taken together, the utility and versatility of the NIR fluorescent smart probe for imaging tumor hypoxia lends itself to (i) future studies involving *orthotopic* murine GBM 39 tumor models and (ii) serving the role of an excellent optical imaging tool that shows great promise for preclinical imaging applications or its translation. In addition, we look forward to future studies seeking to (i) utilize PET imaging for multiplexing and/or (ii) concurrently employ oxygen‐sensitive microelectrodes to quantitatively determine and correlate oxygenation levels to obtained NIR fluorescent TBRs when using **NO**_**2**_ **‐Rosol**.

## AUTHOR CONTRIBUTIONS

**Kenneth S. Hettie:** Conceptualization; data curation; formal analysis; funding acquisition; investigation; methodology; project administration; supervision; validation; writing‐original draft; writing‐review & editing. **Jessica L. Klockow:** Data curation; funding acquisition; investigation; methodology; writing‐original draft. **Eui Jung Moon:** Data curation; formal analysis; investigation; methodology. **Amato J. Giacca:** Resources. **Frederick T. Chin:** Funding acquisition; methodology; resources.

## CONFLICT OF INTERESTS

The authors have stated explicitly that there are no conflicts of interest in connection with this article.

## ETHICAL STATEMENT

All maintenance, handling, monitoring, and experimental procedures were performed in accordance to a protocol that was approved by The Administrative Panel on Laboratory Animal Care (APLAC) of Stanford University, wherein such approved protocol and committee comply with all federal and state regulations governing the humane care and use of laboratory animals as well as the National Institutes of Health guide for the care and use of lLaboratory animals (NIH Publications No. 8023, revised 1978). Institutional approval and consent (patient or next of kin) for the use of patient‐derived cell lines was obtained.

## Data Availability

Research data are not shared.
